# Altitude-related cough

**DOI:** 10.1186/1745-9974-9-23

**Published:** 2013-10-31

**Authors:** Nicholas P Mason

**Affiliations:** 1 Consultant in Critical Care Medicine, Royal Gwent Hospital, Newport NP20 2UB, UK

## Abstract

Altitude-related cough is a troublesome condition of uncertain aetiology that affects many visitors to high altitude. The traditionally held belief that it was due solely to the inspiration of cold, dry air was refuted by observations and experiments in long duration hypobaric chamber studies. It is likely that altitude-related cough is a symptom of a number of possible perturbations in the cough reflex arc that may exist independently or together. These include loss of water from the respiratory tract; respiratory tract infections and sub-clinical high altitude pulmonary oedema. The published work on altitude-related cough is reviewed and possible aetiologies for the condition are discussed.

## Introduction

‘Alan was still racked by frequent coughs, and periodically, as if by auto-suggestion, I found that I too was succumbing to a bout. Once started, there was no escape. The cold dry air compounded the irritation in the throat and the victim’s body would be shaken by the hacking cough until randomly flung free of its spell. The nights at base camp as well as on the mountain were often punctuated by staccato bursts of noise disturbing the sleep of the sufferer and all those around’.

This description by the late Joe Tasker is typical of the debilitating, paroxysmal cough that is well known within the mountaineering community to effect visitors to high altitude [1-3]. Four members of the 1971 International Himalayan Expedition to Mount Everest suffered rib fractures as a result of coughing and other similar accounts of cough-related rib fractures at high altitude exist [2-4]. In a survey of 283 trekkers walking in the Everest region of Nepal, 42% suffered from cough while in another study, again in the Everest region of Nepal, the prevalence of cough was found to be 22% between 4243 and 4937 m [5].

**Figure 1:**
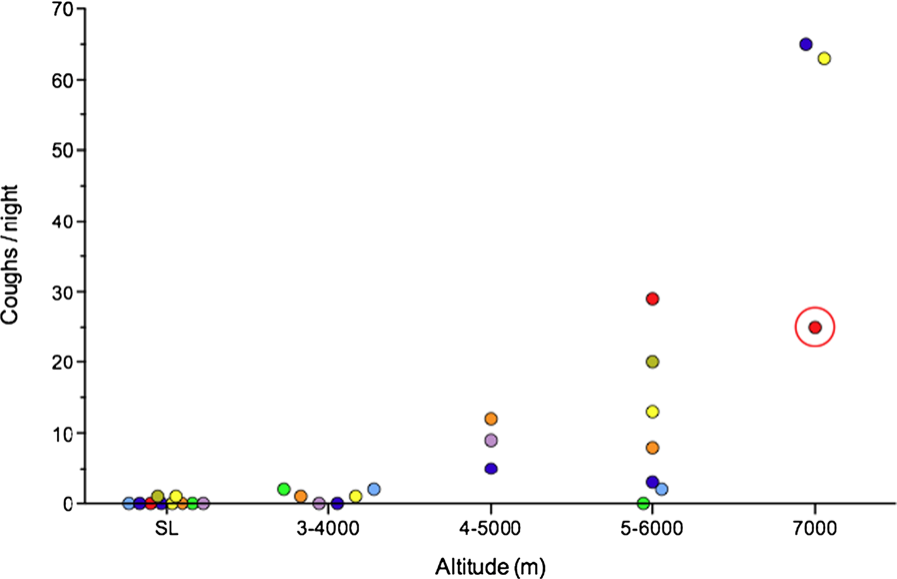
**Nocturnal cough frequency in 10 subjects during a trek to Mount Everest Base Camp, Nepal (5300 m), including 3 climbers in whom recordings were made at 7000 m on Mount Everest.** Recordings were only possible in 3 subjects at between 4 and 5000 m due to logistical problems. The circled data point represents a climber in whom the recorder suffered battery failure after only 1 hour. Data plotted from [6].

The first formal study of cough at high altitude took place during the 1994 British Mount Everest Expedition when 10 subjects trekking from 2800 m to Everest Base Camp at 5300 m underwent nocturnal cough frequency monitoring [6]. Nocturnal cough frequency increased with increasing altitude. In three climbers in whom recordings were made at 7000 m on Everest there was a massive increase in nocturnal cough frequency (Figure 1). As part of the same study citric acid cough threshold was measured in 42 subjects at sea level and on arrival at Base Camp at 5300 m and again at Base Camp in 23 of these same subjects after they had spent at least 9 days at or above 5000 m. Citric acid cough threshold was unchanged on arrival at Base Camp compared with sea level but was significantly reduced on the second visit to Base Camp compared with both the sea level and first visit to Base Camp measurements. Despite both anecdotal and observational evidence of an increase in cough with altitude, and a demonstrated change in citric acid cough threshold, the aetiology of altitude-related cough is poorly understood and treatment remains unsatisfactory.

### Aetiology

Altitude-related cough has traditionally been attributed to the inspiration of the cold, dry air which characterises the high altitude mountain environment [2]. Interestingly however, the author is not aware of any similar reports in the polar exploration literature where exercising explorers are exposed to a similar environment of cold, dry air, but often at a considerably lower altitude. During Operation Everest II eight subjects who underwent a simulated ascent of Mount Everest in a hypobaric chamber were decompressed over a 40 day period to a barometric pressure of 253 mm Hg (33.7 kPa) equivalent to the summit of Everest (8848 m). At altitudes above 7000 m the majority of the subjects developed pain and dryness in the throat and an irritating cough, despite the chamber being maintained at a relative humidity of between 72 and 82% and a temperature of 23°C [7]. This brought into question the widely held view that cough at high altitude was due to the inspiration of dry, cold air.

**Figure 2:**
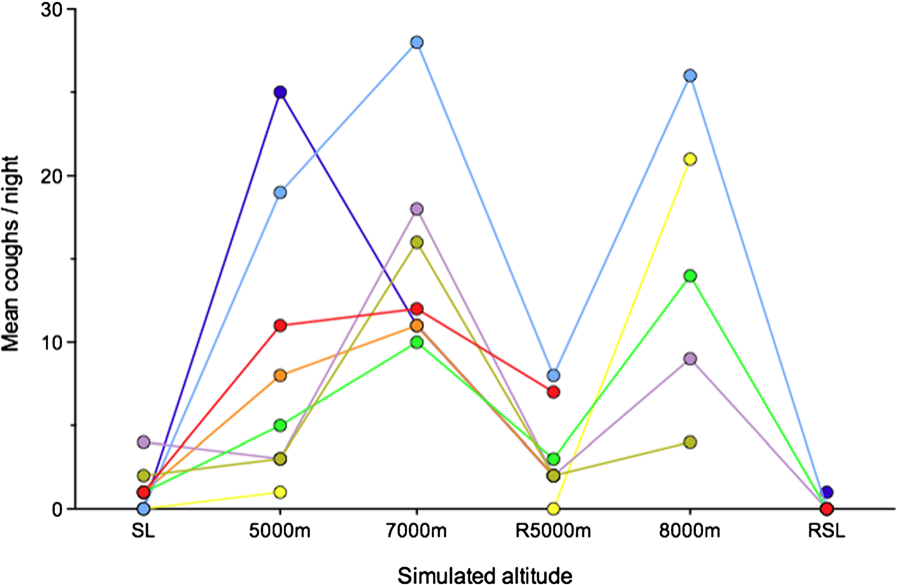
**Nocturnal cough frequency in the eight subjects taking part in Operation Everest III.** R5000m: Return to 5000 m, RSL: Return to Sea Level. Data plotted from [8].

In the next major hypobaric chamber study, Operation Everest III, during which 8 subjects again underwent a simulated ascent of Mount Everest, Mason et al. [8] studied nocturnal cough frequency and citric acid cough threshold over the 40-day period of the study. During the experiment the temperature and relative humidity of the chamber were maintained at between 18 and 24°C and 30-60% respectively. Nocturnal cough frequency increased with increasing altitude and when the subjects descended to 5000 m to recuperate before the ascent to the “summit,” the cough frequency fell before increasing again on ascent to 8000 m. Cough frequency immediately returned to control values on descent to sea level. This data is shown in Figure 2.

Citric acid cough threshold was measured at sea level, 5000 m, the return to 5000 m and at 8000m. There was no difference between either 5000 m or the return to 5000 m and the sea level control, which may have been due to inadequate study power. A maximum of 8 people could be accommodated in the hypobaric chamber, whereas power calculations based on previous work [6] suggested that at least 9 subjects would be required to detect a significant fall in cough threshold. Cough threshold was however reduced at 8000 m compared to both sea level and the first arrival at 5000 m, despite the constant environmental humidity and temperature. The results of Operation Everest III would thus appear to refute the idea that altitude-related cough is due to the inspiration of dry, cold air.

If altitude-related cough is not simply due to the inspiration of dry, cold air, what might its aetiology be? There are a number of potential mechanisms. Each will be considered in turn:

– Acute mountain sickness (AMS).

– Sub-clinical high altitude pulmonary oedema.

– Changes in the central and peripheral control of cough.

– Loss of water from the respiratory tract.

– Respiratory tract infections.

– Bronchoconstriction and asthma.

– Vasomotor-rhinitis and post-nasal drip.

– Gastro-oesophageal reflux.

### Acute mountain sickness

Acute mountain sickness (AMS) is a common condition that effects healthy individuals on ascent to altitude. The incidence of AMS is dependent upon both the altitude and speed of ascent [9]. Rarely people may be affected at altitudes as low as 2000–2500 m while at altitudes between 3500 and 4500 m the reported incidence varies between 30 and 45% [10]. Symptoms normally appear several hours after arrival at a new altitude and consist of a headache and at least one other symptom from fatigue, dizziness, gastrointestinal disturbance and difficulty sleeping. Peripheral oedema is a common finding [11]. The symptoms, which are probably due to mild cerebral oedema, may be regarded as signs of slow acclimatization and normally will disappear after 2–3 days at a given altitude, although they may reappear on ascent to a new altitude. In severe cases the boundary between AMS and high altitude cerebral oedema (HACE) is blurred.

Despite both AMS and cough occurring commonly at high altitude, there was no demonstrable relationship between AMS and cough in any of the published papers [6,8,12,13]. In subjects with isolated AMS cough is a rarely reported symptom [9,11]. In 21 studies dealing with AMS cough was not reported as a problem [14]. The inability to demonstrate a relationship between altitude-related cough and AMS may reflect the inability of the Lake Louise Scoring system [15], the standard tool used to measure the presence and severity of AMS, to detect early AMS. On balance however it seems unlikely that AMS plays a significant role in the aetiology of altitude-related cough.

### Sub-clinical high altitude pulmonary oedema

High-altitude pulmonary oedema (HAPE) is a non-cardiogenic, hydrostatic pulmonary oedema which occurs predominantly in unacclimatised individuals up to 72 hours after ascent to a new altitude. The prevalence depends upon the altitude, the rate of ascent and individual susceptibility. Some individuals are susceptible to developing the condition repeatedly on ascent to altitude [16]. While these HAPE-susceptible individuals are frequently studied they may not be representative of the general population. The characteristic pathophysiological finding in patients with HAPE is an abnormally elevated pulmonary arterial pressor response to hypoxia with pulmonary artery systolic pressures of around 60–80mmHg, although values in excess of 140 mmHg have been reported [16,17]. Reduction in this acute pulmonary hypertension forms the mainstay of pharmacological therapy, used to facilitate the definitive treatment: descent.

Although only a minority who go to high altitude develop HAPE, some workers hypothesise that the majority of people ascending to altitude may develop sub-clinical pulmonary oedema. Evidence cited in favour of this argument includes the fall in forced vital capacity (FVC) on ascent to altitude [18]; changes in the phase III slope (the alveolar plateau) of the nitrogen washout curve [19,20] and clinical, radiological [21,22] and electrical impedance tomography [23] changes consistent with an increase in extra-vascular lung water, even in apparently asymptomatic subjects. This view however is not universally held [24].

The apparently simple question question as to whether altitude-related cough could be a manifestation of sub-clinical pulmonary oedema reveals the ongoing uncertainty that exists regarding the identity of the afferent pathways responsible for cough. It is still unclear as to whether pulmonary oedema can cause cough in humans. If it can, is there a sufficient stimulus in asymptomatic subjects with subclinical pulmonary oedema at altitude to activate such a pathway? Cough is not a common clinical feature in cardiogenic pulmonary oedema. The main anatomical sites that are responsible for cough are located above the segmental airways (division 4) [25,26]. This is significantly more proximal than the airways in which pulmonary oedema would begin to form [27]. Evidence exists however from experiments in rabbits that even small changes in left atrial pressure of between 2 and 5mmHg result in sufficient pulmonary venous congestion as to stimulate airway rapidly adapting receptors [28-30]. It is not known what these pressure changes equate to in humans but it is easy to imagine that pulmonary artery pressures of between 35 and 45 mmHg which have been reported in healthy control subjects free of HAPE [31,32] could result in comparable pulmonary venous congestion as an increase in left atrial pressure of 2–5 mmHg.

The natural history of HAPE, which is associated with a recent gain in altitude and improves dramatically with descent, is similar to the cough seen in Operation Everest III [8]. However it is the author’s experience that altitude-related cough can also be present throughout a prolonged stay at one altitude when acclimatisation would be well advanced and may persist for a time after descent even to sea level. If altitude-related cough was due to pulmonary oedema, one might expect HAPE-susceptible subjects to have more severe cough. This does not appear to be the case. While it is attractive to speculate that sub-clinical pulmonary oedema causes altitude-related cough, further work, including interventional studies to assess the impact of a reduction in pulmonary artery pressure on altitude-related cough, is required before this hypothesis can be confirmed or refuted.

### The central control of cough

Respiratory control undergoes profound changes with acclimatisation to high altitude [33]. The central control of cough is complex [34-36] but there are a number of factors which suppress cough that also suppress ventilation, such as sleep [37,38] and centrally acting anti-tussive agents [39]. Banner [40] demonstrated a relationship between the hypercapnic ventilatory response (HCVR) and the cough threshold to hypotonic saline, with those subjects who responded to the hypotonic saline challenge having a higher HCVR than the subjects who did not respond. In addition *Post*-*hoc* analysis of data from the 1994 British Mount Everest Medical Expedition demonstrated a relationship between the citric acid cough threshold and the dynamic ventilatory response to CO_2_[41].

**Figure 3:**
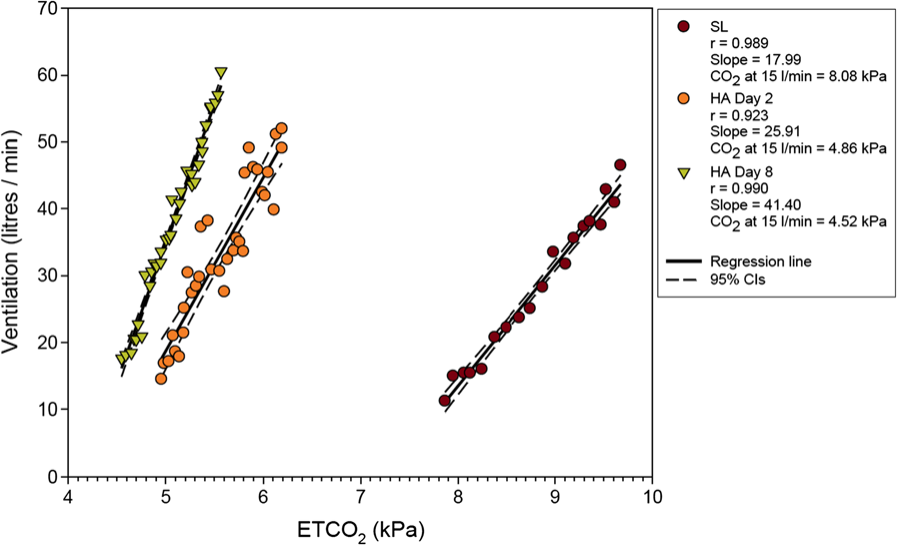
**Sample HCVR plot from subject at sea level and after 2 and 8 days at 5800m.** Data from [13]. CVR is quantified by the slope S and the end-tidal PCO_2_ for a ventilation of 15 l/min.

As a result of these findings Thompson et al. [13] studied the relationship between the citric acid cough threshold and the hypercapnic ventilatory response (HCVR) in 25 healthy subjects during a 9 day stay at 5200 m. Citric acid cough threshold fell significantly on ascent to altitude and the HCVR, measured using the Read re-breathing method and expressed by the slope S, increased significantly on ascent to 5200 m. This data is shown in Figure 3. There was however no demonstrable meaningful relationship between the citric acid cough threshold and HCVR, or any change in these parameters on ascent to altitude. It is therefore unlikely that altitude-related cough is mediated through changes in central control mechanisms.

### Bradykinin and altitude-related cough

While the neuronal pathways that mediate the citric acid cough threshold remain debated they include stimulation of airway sensory nerves including the rapidly adapting receptor (RAR) [42]. Cough is a well recognised side effect in a proportion of patients taking angiotensin converting enzyme (ACE) inhibitors and is thought to be due to sensitisation of airway RARs by increased levels of plasma bradykinin and substance P [43]. Bradykinin is degraded by a number of enzymes collectively known as kininases which include ACE, aminopeptidase P and neutral endopeptidase and in human serum over 75% of bradykinin metabolism occurs via ACE [44,45]. The early literature on the response of serum ACE activity to hypoxia in humans is confusing and contradictory [46]. Nothing was known about what happened to bradykinin at altitude beyond exposure to 1 hour of normobaric hypoxia [47].

Mason et al. [12] measured nocturnal cough frequency, citric acid cough threshold, serum angiotensin converting enzyme activity and plasma bradykinin concentration in 20 healthy volunteers before and during a stay at 3800 m. Citric acid cough threshold fell on ascent to 3800 m; serum ACE activity was unchanged although plasma bradykinin fell precipitously. It is not possible to measure lung ACE activity in living human subjects although measurement of whole lung and serum ACE activity in dogs during exposure to chronic hypoxia yielded comparable results [48]. Understandably no comparable data exists in humans and the most likely explanation for these findings is that serum ACE activity did not parallel an increase in local tissue ACE activity in the pulmonary endothelium [49]. Alternatively it is possible that bradykinin was metabolised by a kininase other than ACE. The kallikrein-kinin system is predominantly tissue based, and tissue levels of bradykinin are known to exceed those in blood, although in rats changes in plasma levels of bradykinin have been shown to parallel changes in the tissues [50]. A further weakness of this study is that although citric acid cough threshold fell, suggesting an increased sensitivity of the afferent cough reflex arc, there was no increase in the clinical incidence of cough as measured by the nocturnal cough frequency. It thus remains difficult to draw any meaningful conclusion about the role that the kallikrein-kinin system may play in the aetiology of altitude-related cough.

### Respiratory water loss

**Figure 4:**
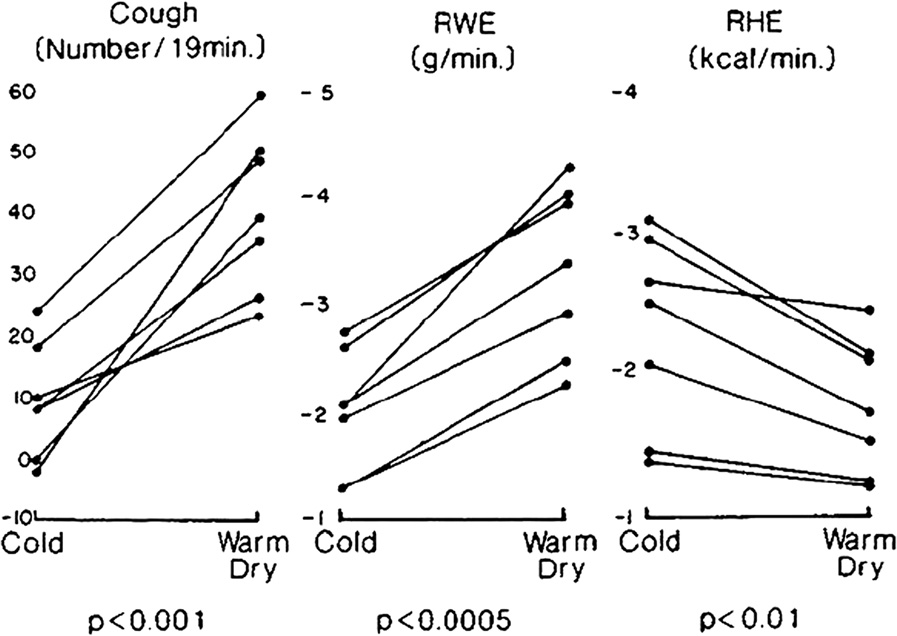
**Number of coughs in excess of baseline (Cough); respiratory water loss (RWE) and respiratory heat loss (RHE) in 7 subjects with exercise-induced cough during a 19 minute period after 4 minutes of hyperpnoea with cold air at -16°C or warm, dry air at 38.7°C.** Both the cold air and the warm dry air had a water content of 0 g m^-3^. From [60].

It is still possible, despite the increase in nocturnal cough frequency and decrease in citric acid cough threshold observed under the controlled environmental conditions of Operation Everest III [8] that water loss from the respiratory tract plays a role in the aetiology of altitude-related cough. Banner et al. [51] studied subjects susceptible to exercise-induced cough and found that hyperpnoea for 4 minutes with cold air, at respiratory rates similar to those occurring with strenuous exercise, was associated with an increase in cough frequency in the 30 minute period post-hyperpnoea. Bronchoconstriction was blocked using inhaled β_2_ agonists and there was no evidence of bronchial hyper-reactivity to a methacholine challenge. In a follow up study, subjects susceptible to exercise-induced cough underwent voluntary isocapnic hyperpnoea to over 90 litres/minute with a variety of inspired air conditions [52]. Cough frequency was found to depend directly upon respiratory water loss in a linear manner and that in the absence of water loss, even in the presence of heat loss, cough frequency did not increase. Hyperpnoea with warm, dry air produced more coughing than hyperpnoea with cold air despite less heat loss. Hyperpnoea with ambient air produced as great an increase in cough frequency as hyperpnoea with cold air because it was associated with significant water loss. These findings are illustrated in Figure 4.

These findings raise the possibility that, at least in a subgroup of sufferers, altitude-related cough may be caused by water loss from the respiratory tract. How this water loss might stimulate afferent cough pathways is not known but possible mechanisms include physical distortion of afferent nerve endings [53] and changes in the ionic composition of airway surface liquid [54]. Increased minute ventilation plays a key role in respiratory acclimatisation to hypobaric hypoxia, increasing with increasing altitude and further still with exercise [33]. It is thus possible that despite controlling the environmental conditions during Operation Everest III [8] respiratory water loss could have contributed to the increase in cough frequency and sensitivity seen. The data on respiratory water loss at altitude is very limited and confined to subjects who were exercising [55]. In the Operation Everest II and III chamber studies the subjects were mainly sedentary.

Other factors may also increase respiratory water loss. There is evidence of subjective nasal blockage and an increase in nasal resistance at altitude which will likely result in increased mouth breathing [56,57]. Water loss is known to increase during mouth, rather than nasal breathing [58].

### Respiratory tract infections

Respiratory tract infections are the commonest cause of acute cough at sea level [59,60]. Signs and symptoms of respiratory tract infection also occur commonly in visitors to altitude [61,62] in whom there is also evidence of impairment of mucociliary transport - a crucial respiratory defence mechanism [56]. There was no clinical evidence of respiratory infection observed in any of the published studies on altitude-related cough [6,8,12,13]. However in the author’s experience cough associated with the production of purulent sputum is not an uncommon finding at altitude, particularly following prolonged exertion. This cough responds poorly to antibiotics. Whether this argues against a bacterial aetiology is unclear although prolonged cough following relatively minor atypical bacterial respiratory infections are well recognised at sea level [63,64]. Viral infections are also a well recognised cause of cough at sea level [65] but no data exists on their prevalence at high altitude. Further work is required on the microbiology of respiratory tract infections in visitors to altitude and their relationship to cough.

### Bronchoconstriction

Cough may be the only presenting symptom of asthma [66,67]. Bronchoconstriction can occur in both asthmatics and healthy subjects on exposure to cold air. The mechanism for this appears to be due to water loss from the respiratory tract rather than a direct effect of temperature on the airways [68]. There was no change in FEV_1_ or PEF associated with the fall in citric acid cough threshold at either Everest Base Camp [6] or during Operation Everest III [8]. Although both acute hypoxia and hypocapnia have been shown to cause bronchoconstriction in animal models [69-71], Pollard et al. [72] could demonstrate no evidence of bronchoconstriction in healthy lowland subjects at Everest Base Camp. Likewise Dehnert et al. [24] demonstrated no change in either effective or specific airway resistance on ascent to 4559 m. It is unlikely that bronchoconstriction plays a major role in altitude-related cough in non-asthmatic subjects.

### Vasomotor rhinitis and post-nasal drip

Upper airway cough syndrome (previously known as post-nasal drip syndrome) is one of the most common causes of chronic cough [73]. Nasal blockage and symptoms of rhinitis are common complaints at altitude [56,61] but their relationship with cough, if any, has not been established.

### Gastro-oesophageal reflux

Gastro-oesophageal reflux has been reported in up to 40% of patients with chronic cough at sea level [74-76] but nothing is known about its prevalence at altitude.

### Changes in nebuliser output at altitude

A potential cause for the change in citric acid cough threshold seen on exposure to hypobaric hypoxia would be an alteration in nebuliser output consequent upon the reduced barometric pressure. Barry studied the output of 3 types of nebulisers in a hypobaric chamber up to an altitude equivalent of 5300 m [77]. The output of salbutamol from both a conventional jet nebuliser and a breath enhanced nebuliser, collected onto a filter, fell by over 50% at simulated altitude compared with sea level, while the output of an ultrasonic nebuliser fell by only 23% at 4200 m and 13% at 5300 m. Subsequently Barry et al. [78] measured serum salbutamol levels in human subjects following nebulised administration of salbutamol via an ultrasonic nebuliser at both sea level and a simulated altitude of 5000 m in a hypobaric chamber. There was no difference in serum salbutamol levels between the 2 altitudes. Providing an ultrasonic nebuliser is used at altitude, as has been the case in all of the published studies [6,8,12,13], it seems unlikely that the increase in sensitivity to citric acid can be explained by alterations in nebuliser output.

### Current understanding of altitude-related cough

Our current understanding of the epidemiology and aetiology of altitude-related cough remains limited. From the published work, to date, the only conclusions that can be drawn are that altitude-related cough exists and its prevalence increases with increasing altitude; it is associated with a decrease in the citric acid cough threshold; is not solely due to the inspiration of dry, cold air, as has traditionally been thought, and the increased sensitivity to inhaled citric acid does not appear to be related to the changes in the central control of ventilation that occur with acclimatisation.

Exploration of the aetiology of altitude-related cough has been limited by the logistical difficulty of carrying out robust research in the high altitude environment [79] or in hypobaric chambers [80]. These difficulties will be only too apparent to those who have worked in this hostile environment and something of a mystery to those who have not. While altitude-related cough undoubtedly exists, one of the problems in the published work, is the lack of a consistent relationship between the sensitivity to inhaled citric acid and the clinical presence of cough, as assessed by nocturnal cough frequency. Nocturnal cough frequency increased on arrival at Everest Base Camp but was not accompanied by a fall in a citric acid cough threshold [6]. Conversely citric acid cough threshold fell at the relatively low altitude of 3800 m but was not accompanied by any change in nocturnal cough frequency [12]. These conflicting results are undoubtedly compounded by the small number of subjects in the published studies which is also the likely explanation of the difficulty in demonstrating a significant change in cough frequency or citric acid cough threshold below altitudes of 7000 m [6,8] despite, anecdotally, cough being a clinical problem at lower altitudes.

It seems most likely that, as at sea level, altitude-related cough is a symptom of a number of possible perturbations in the cough reflex arc that may exist independently or together. The most likely aetiological factors are infection or water loss from the respiratory tract leading to trauma of the respiratory mucosa and tracheo-bronchitis, and sub-clinical pulmonary oedema. Respiratory water loss and sub-clinical pulmonary oedema could both be precipitated or worsened by exercise. Future work should investigate the role of these aetiologies as well as quantifying the prevalence of altitude-related cough in larger populations at altitude and to correlate better the relationship between clinical cough and inspired tussive challenges.

## Competing interests

The author declares that he has no competing interests.
